# Assessing the impact of human mobility to predict regional excess death in Ecuador

**DOI:** 10.1038/s41598-021-03926-0

**Published:** 2022-01-10

**Authors:** Leticia Cuéllar, Irene Torres, Ethan Romero-Severson, Riya Mahesh, Nathaniel Ortega, Sarah Pungitore, Ruian Ke, Nicolas Hengartner

**Affiliations:** 1 Los Alamos National Laboratory, Los Alamos, NM USA; 2 Fundación Octaedro, Quito, Ecuador

**Keywords:** Public health, Epidemiology, Infectious diseases

## Abstract

COVID-19 outbreaks have had high mortality in low- and middle-income countries such as Ecuador. Human mobility is an important factor influencing the spread of diseases possibly leading to a high burden of disease at the country level. Drastic control measures, such as complete lockdown, are effective epidemic controls, yet in practice one hopes that a partial shutdown would suffice. It is an open problem to determine how much mobility can be allowed while controlling an outbreak. In this paper, we use statistical models to relate human mobility to the excess death in Ecuador while controlling for demographic factors. The mobility index provided by GRANDATA, based on mobile phone users, represents the change of number of out-of-home events with respect to a benchmark date (March 2nd, 2020). The study confirms the global trend that more men are dying than expected compared to women, and that people under 30 show less deaths than expected, particularly individuals younger than 20 with a death rate reduction between 22 and 27%. The weekly median mobility time series shows a sharp decrease in human mobility immediately after a national lockdown was declared on March 17, 2020 and a progressive increase towards the pre-lockdown level within two months. Relating median mobility to excess deaths shows a lag in its effect: first, a decrease in mobility in the previous two to three weeks decreases excess death and, more novel, we found an increase of mobility variability four weeks prior increases the number of excess deaths.

## Introduction

The coronavirus disease (COVID-19) pandemic has a high morbidity and mortality. Ecuador, like many Latin American countries^1^, has been hit hard by the COVID-19 pandemic, with over 242 thousand reported cases and 14,500 deaths by the end of January 2021. The first confirmed case of COVID-19, reported on February 29, 2020, was a woman in her ‘70s who returned from Spain two weeks prior^2^. On March 13, with 23 confirmed cases, that same woman became the first COVID-19 confirmed fatality^3^. By April, Ecuador emerged as an "epicenter" of the pandemic in Latin America, with reports of uncollected dead bodies remaining for days in the streets^4^. To control the outbreak, schools and universities in Ecuador were closed on March 13th ^5,6^, and on March 17th, Ecuador implemented a national lockdown^7^. Both of these measures decrease the spread of the disease by reducing contacts between infected and susceptible individuals. It is possible to measure the compliance to these orders by tracking human mobility derived from cell phone data. Such data has emerged as a useful tool to measure human mobility and its relationship to the spread of diseases^8,9,10^ including SARS-COV-2^8,11,12^, malaria^13,14^ cholera^15^, measles^16^, dengue^17,18^, and Ebola^19,20^.

Quantifying the severity of the outbreak in Ecuador is challenging. Due to limited testing, the reported daily counts of COVID-19 incidence and death underestimate the true magnitude of the outbreak. Excess death, which compares total number of observed deaths to the expected number of deaths, is commonly used to assess official undercounted burden of an infectious disease. From a health care and societal perspective, quantifying the excess death associated to an epidemic is informative^21–23^ and is the most reliable measure of current COVID-19 data available^24^. Analysis of the increase in all-cause mortality can complement the more traditional analysis of the time series of disease incidence and disease-related deaths.

**Figure 1:**
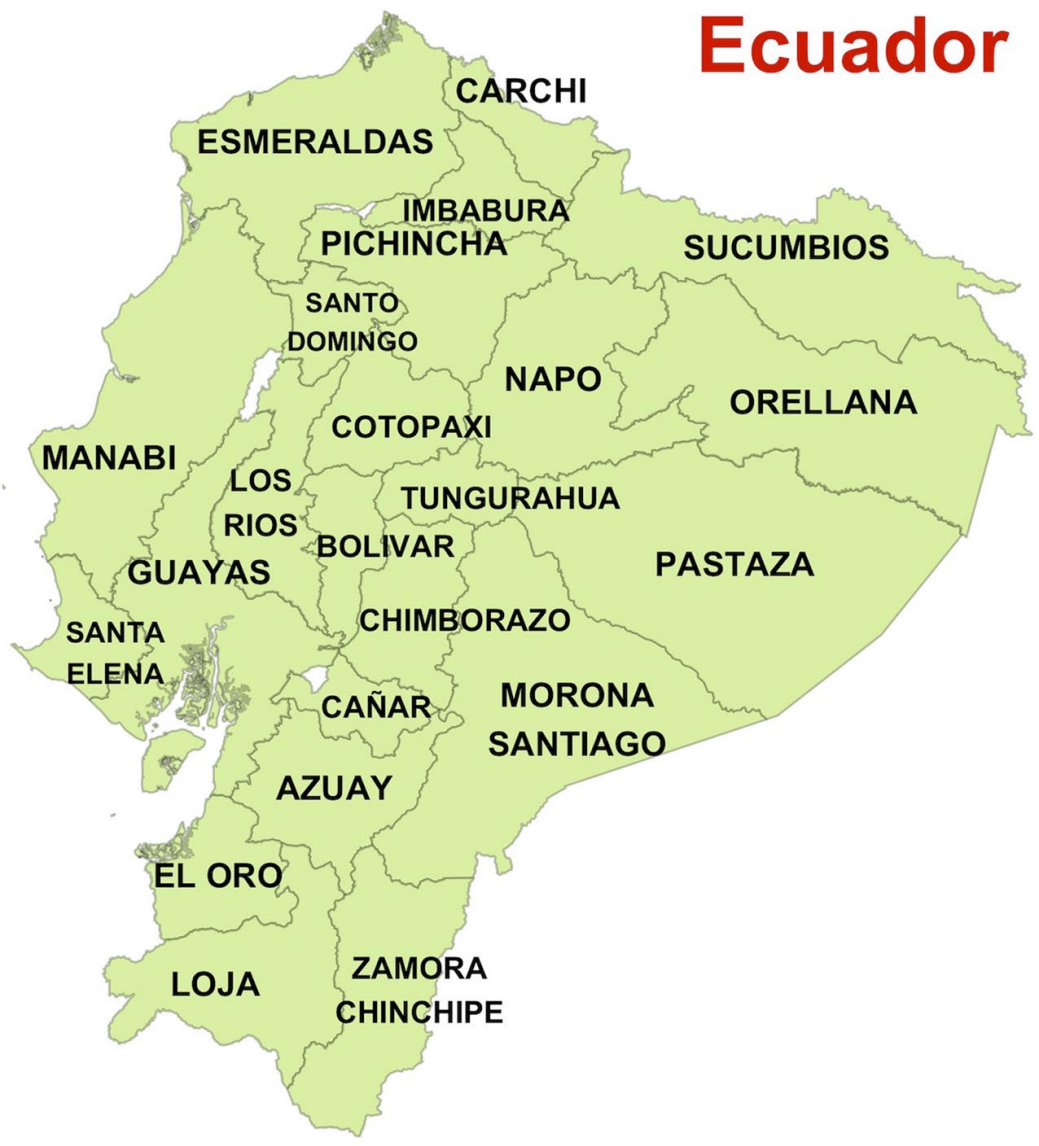
Map of Ecuador with provinces Figure created in R version 4.0.3 (https://www.r-project.org).

In this paper, we empirically address the hypothesis that a reduction in mobility predicts a decrease in the future number of COVID-19 cases and deaths. We relate the temporal dynamic of excess deaths within each of the 24 provinces in Ecuador (see Fig. 1) to human mobility characteristics derived from mobile phones data provided by GRANDATA through the United Nations Development Program. We show that the implementation of lockdown can dramatically reduce the risk of excess deaths especially for provinces that were experiencing surges of SARS-CoV-2 outbreak (e.g. Guayas and Santa Elena). For other provinces, lockdown prevented and delayed the wave of excess deaths by several months (e.g. Pichincha).

We demonstrate that both, the median mobility and the variability of mobility as measured by the Interquartile Range (IQR), are statistically significant explanatory variables for excess deaths; that is, that the effect of lockdowns are mediated, at least partially, through reduction in individual-level mobility. However, the mobility data lacks demographic covariates, limiting its predictive power given the importance of demographic characteristics in risk of both COVID-19 infection and death. Given the usefulness of mobility data to predict epidemic progression, we suggest that future collection of mobility data have demographic covariates to increase the predictive power of predictive models. This paper is organized as follows: Sect. 2 provides background information on Ecuador and the need to relate population mobility to excess deaths. Section 3 describes the mobility data and the death records used to estimate excess deaths. Section 4 describes the statistical methods whose results are shown in Sect. 5 and discussed in Sect. 6. We conclude in Sect. 7.

## Background

### Ecuador

Ecuador has an estimated population of 17.5 million; it is located by the Pacific Ocean, at the northwest of South America, and borders with Colombia to the north and Peru to the south and east. It is divided into 24 provinces, that are each further divided into cantons. The World Bank classifies it as an upper middle-income country, with a Gini inequality coefficient of 45.4 (where 100 is maximum inequality).

Ecuador’s healthcare system is patchwork of public and private healthcare providers. The public/private health insurance covers 40%/60% of the (employed or farmer) population and the Ministry of Public Health services for the uninsured. At least 39% of total health spending in Ecuador is out-of-pocket^25^ with more than half of this amount destined for medication. Life expectancy at birth for women is 79.3 and for men, 73.9 years^25^ which ranks 3rd best among the 12 South American countries in 2019.

Ecuador officially registered a relatively small number of COVID-19 deaths; yet news articles from March and April 2020 report a large number of deaths. Estimates of excess deaths in^26,27^ are consistent with Ecuador having a higher than officially reported death toll.

### Excess deaths in Ecuador

Our previous study revealed the true impact of COVID-19 on mortality in Ecuador^27,28^ by quantifying the excess deaths in Ecuador by province, sex, age and ethnicity. Our key finding are a 70% more deaths than expected from January 1st to September 23rd, 2020, which is 3 times the level of excess deaths found in high income countries like the United States^22,29,30^, England and Wales^21^, and Italy^31,32^. Strikingly, the Provinces of Guayas and Santa Elena, the worst affected by the pandemic, have over 200% more deaths than expected, with the highest peaks in late March and early April reaching between a 12 to 15-fold more deaths than expected. As for demographics, we found similar patterns to those observed in other countries, for example we found that men had a death rate 183% of the expected value, while the rate in women was 153% of expected deaths; and even though we find that excess death increased with age, the group mostly affected is the [60, 69] age group with a death rate 233% of the expected value, while the age group with people older than 80 had a death rate 160% of the expected level. Finally, we found that the indigenous ethnic group was disproportionally affected with a death rate of 220% more than expected compared to 136% for the mestizo’s group, the most prevalent ethnic group in Ecuador.

### Strict lockdown in Ecuador

To control the outbreak, Ecuador closed schools on March 13th, implemented a national lockdown on March 17th and instituted mandatory face masks from April 7, 2020^33^. The lockdown limited all non-essential movement, including halting intra and inter-provincial public and private transportation except for medical and legal personnel, and police and military forces. From May 11, 2021, cantons progressively opened up on an individual basis, depending on the number of cases reported. This involved public transportation, restaurants and stores with limited occupancy. Schools and universities remained closed during the entire study period.

### Relating excess deaths to population mobility characteristics

Using the methodology from^27,28^, we calculate time series of weekly all cause Excess Death Factor (EDF), the ratio of observed over expected deaths, in each province stratified by age group and sex. Spatial–temporal analysis of these time series of the weekly reveals a wave of COVID that originated in Guayas and spread through the rest of Ecuador over a period of six months, from March 2020 to August 2020. This suggests that Ecuador suffered from a single outbreak with a complex spatiotemporal pattern rather than two distinct waves.

Physiologically, the dynamics of excess deaths time series depends on the number of infected individuals in previous weeks. The latter depends on many factors, including human behavior related to the mixing between infected and susceptible individuals. We expect that behavior change during and after the strict lockdown will have a delayed impact on the dynamic of excess deaths. In this paper, we hypothesize that the magnitude of the epidemic (as measured by excess deaths) depends on characteristics of past population mobility distributions. Specifically, we relate weekly excess deaths to mobility characteristics derived from mobile phone data provided by GRANDATA through the United Nations Development Program to quantify compliance with mandatory lockdown orders in Ecuador, and changing mobility patterns after the strict lockdown is lifted.

Cellular phone mobility data are readily available, and can be aggregated at relevant geographical resolution. In our case, we have mobility data for each Ecuadorian canton. While this type of data can be collected systematically, aggregated mobility data may be of limited use as a predictor of disease spread. For example, the data lacks demographic characteristics of the users. Since age and sex are important predictors for excess death^27,28,34,35^ having the mobility data disaggregated similarly would be useful to understand how mobility^36,37^ impacts excess death. Further, the GRANDATA excludes people with limited activity, essentially excluding proportion of individuals staying home, including those telecommuting. Nevertheless, the data from GRANDATA are a direct measure of the relationship between government restrictions, mobility, and invaluable to quantify compliance to mobility restrictions imposed to mitigate the spread of COVID.

## Description of the data

### Mobility data

GRANDATA is a San Francisco-based company that leverages advanced research in Human Dynamics to identify market trends and predict customer actions. They made their data from Latin America and the Caribbean available to the United Nations Development Program (UNDP) to help combat COVID-19 in those regions^38^. UNDP provided limited access to the data through competitive research proposal evaluation^39^.

GRANDATA provided mobility indexes for 191 cantons (out of the 218) with no data for 27 cantons from March 1st, 2020 to November 1st, 2020. The indices were obtained as follows: mobile phone users, with their consent, were geolocated to track their mobility patterns, using the most frequent location (the mode of that distribution) as their place of residence. The daily number of “out-of-home” events (without keeping track of the destination of each event) were aggregated to preserve the anonymity of phone users and reported as percentage difference for daily number of events relative to the baseline date of March 2nd, 2020. While our data does not have the richness of mobility flow data, it provides insights into human behavior and compliance to strict lockdown, and is sufficiently informative to predict excess deaths.

**Figure 2:**
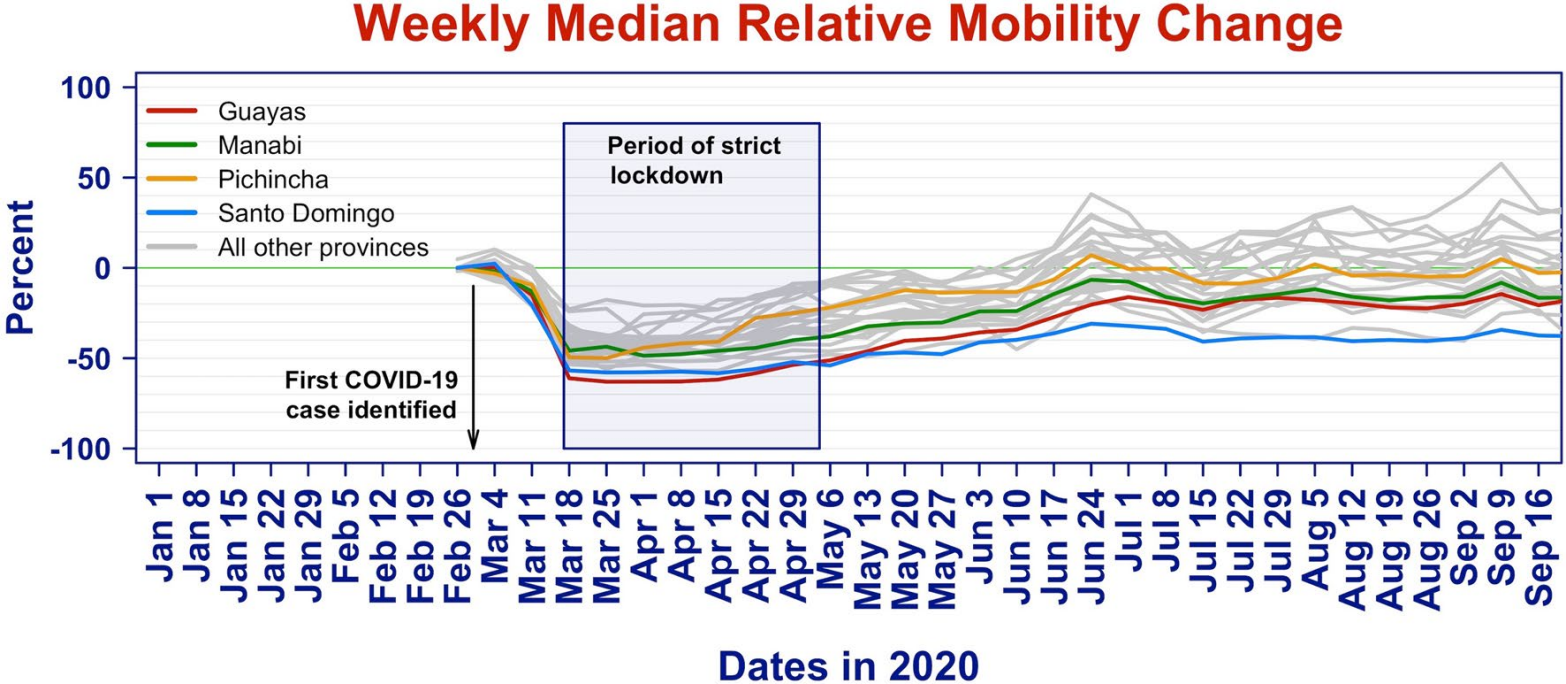
Time series of relative median mobility change for each province.

For each province, we compute the population weighted median, population weighted inter-quartile range, and a population weighted measure of skewness. Figure 2 displays the time series of the median mobility for each province, highlighting in color the statistics of the provinces Guayas, Manabí, Pichincha, and Santo Domingo. The box indicates the period of strict lockdown. A relative change of -0.5 indicates that there are half as many "out-of-home" travels, a value of 0 corresponds to normal out of home travel, and a positive value of 0.5 correspond to a 50% increase in the number of out-of-home trips.

**Figure 3:**
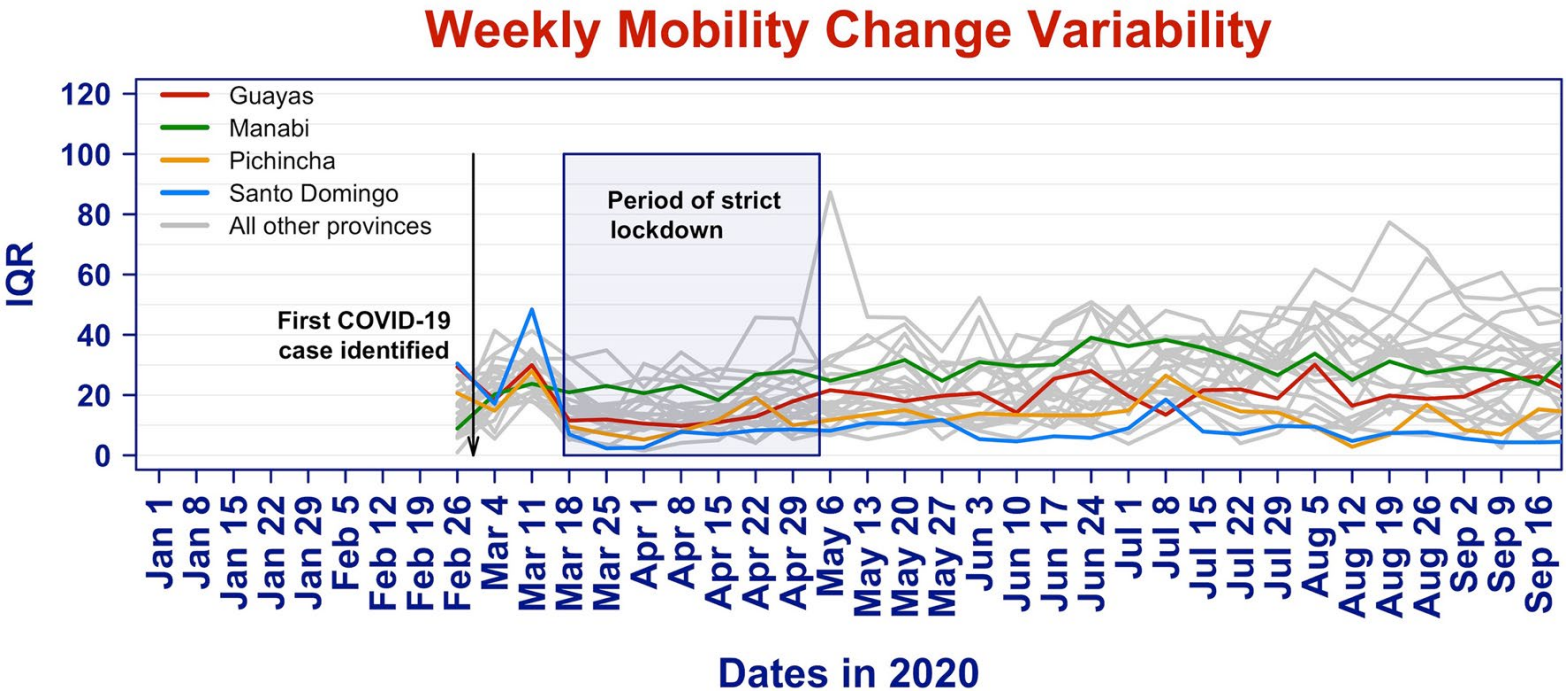
Time series of mobility change variability (IQR) for all Ecuadorian provinces.

Our hypothesis is that the entire mobility distribution, and not just the median or mean, impact the spread of COVID-19. We summarize the variability of the mobility scores by the Inter-Quartile Range (IQR). The IQR measures the spread of a distribution by taking the difference between the 75% quantile and the 25% quantile. For a Gaussian distribution, the IQR is 1.35 times the standard deviation. Figure 3 displays the time series of IQR in each province in gray, with the values for the provinces of Guayas, Manabí, Pichincha, and Santo Domingo highlighted in color. The box indicates the period of strict lockdown.

### Death data

Historical all-cause mortality records from 2015 to 2019 were obtained from the Ecuadorian National Institute of Statistics and Census. Individual records include date of death, age, sex, and ethnicity of the diseased, place of death registration, residence, and the International Classification of Disease (ICD) code for the cause of death. The Ecuadorian Ministry of Government provided death records from all causes that occurred from January 1st, 2020 to September 26th, 2020. In addition to the date of death, records report sex, age, registration, and residence location by parish, canton, and province, but *without the cause of death*.

For our analysis, we computed the number of death per week, sex, age, and province. We binned age into nine 10-year age groups ([0,9], [10,19], …, [70, 79] and [80 and older]). For all years (2015–2020), week 1 was set from January 1 to January 7, week 2 from January 8 to January 14, and so on. In all cases, week 53 contains less than 7 days. We calculated counts for all 24 Ecuadorian provinces but ignored the three smaller "Not Delimited Areas". We use the 2020 population estimates from the INEC (the Ecuadorian National Institute of Statistics and Census).

Individual records of COVID-19 incidence and testing from Ecuador were obtained from the Ecuadorian Ministry of Public Health. Death records were obtained from the Ministry of Interior. All records were aggregated at the weekly level and binned by sex, age group, and province. Ethics committee approval for the use of health patient information and death records was obtained from the Ethical Committee for Expedited Review of COVID-19 Research of the Ecuadorian Ministry of Health. The study has been approved by the Human Subjects Research Review Board, Los Alamos National Laboratory.

## Methods

### Estimation of excess death

We fitted a Poisson log-linear model to the baseline death counts using the *glm* function in R 4.0.3 on the historic 2015–2019 data. The predictor variables included sex, age group and their interactions, province and week of the year as a factor. Using this fitted model, we predict the time series of weekly expected number of deaths from all causes in each province, disaggregated by age and sex. The fitting of this model was described in^27^.

The excess death is the difference between the observed number of deaths in 2020 and the model predicted expected number of deaths; that is, the number of deaths above what would have been expected in 2020 were it a typical year. The Excess Death Factor (EDF) is the ratio of the observed over the expected number of deaths. The EDF implicitly normalizes each province by its population size, allowing them to be compared across provinces. An EDF of 2 means that there are twice as many deaths during the pandemic than in a normal year. The presumption is that these deaths are attributed to COVID-19, although there is uncertainty in that attribution. Indeed, it is possible that mortality from all causes is higher because the healthcare system is overwhelmed, or lower because the strict lockdown likely prevented some deaths (e.g., by reducing the number of vehicle accidents). However, the EDF gives a much clearer image of COVID-19 associated mortality than the confirmed COVID deaths.

### Statistical analysis

Given that the outbreak in each province reaches its peak in different weeks, we chose to model each province separately. On average, a death from COVID-19 is estimated to occur two to eight weeks after infection^40^. To explore the effect of this delay, we computed the cross-correlation between the time series of the EDF and the mobility statistics. That is, we calculated the correlation between the EDF in a given week, and the mobility statistics in prior weeks. While a correlation does not imply causation, the cross-correlation shows how changes in mobility in the past impact today’s EDF.

We used Poisson log-linear regression to explain the EDFs by mobility, while controlling for age and sex. To do so, we model the observed weekly death Y in each province as Poisson distributed variable with mean

$$\mathrm{log}E[Y]=\mathrm{log}\left(\mathrm{predicted}\right)+\sum {\mathrm{\alpha }}_{\mathrm{j}}{W}_{j}$$

where W_1_,…, W_k_ are the explanatory variables in the model, and log(predicted) is the logarithm of the predicted baseline for the number of deaths based on the historical data. The latter is used as an offset in the regression. To account for the delay between mobility change and death, our model included lagged mobility statistics, that is, we only included mobility statistics from the 2, 3, and 4 previous weeks. Further lags were not considered for the regression analysis given the limited number of weeks for the mobility data.

We applied model selection with stepwise regression using the Akaike information criterion (AIC) criterion on the mobility statistics to identify which lags and other summary statistics were important. We note that the AIC is permissive in that it may retain a few variables that are only weakly associated with the response.

Finally, we compute a measure of variability explained by the mobility statistics by reporting the ratio of the difference of the deviance of the fitted model with and without the mobility statistics, divided by the difference of the full model^41^ and the model with only the demographic variables. This quantity is similar in spirit to the R^2^ statistic in standard linear regression.

## Results

### Geographical distribution of excess death

**Figure 4:**
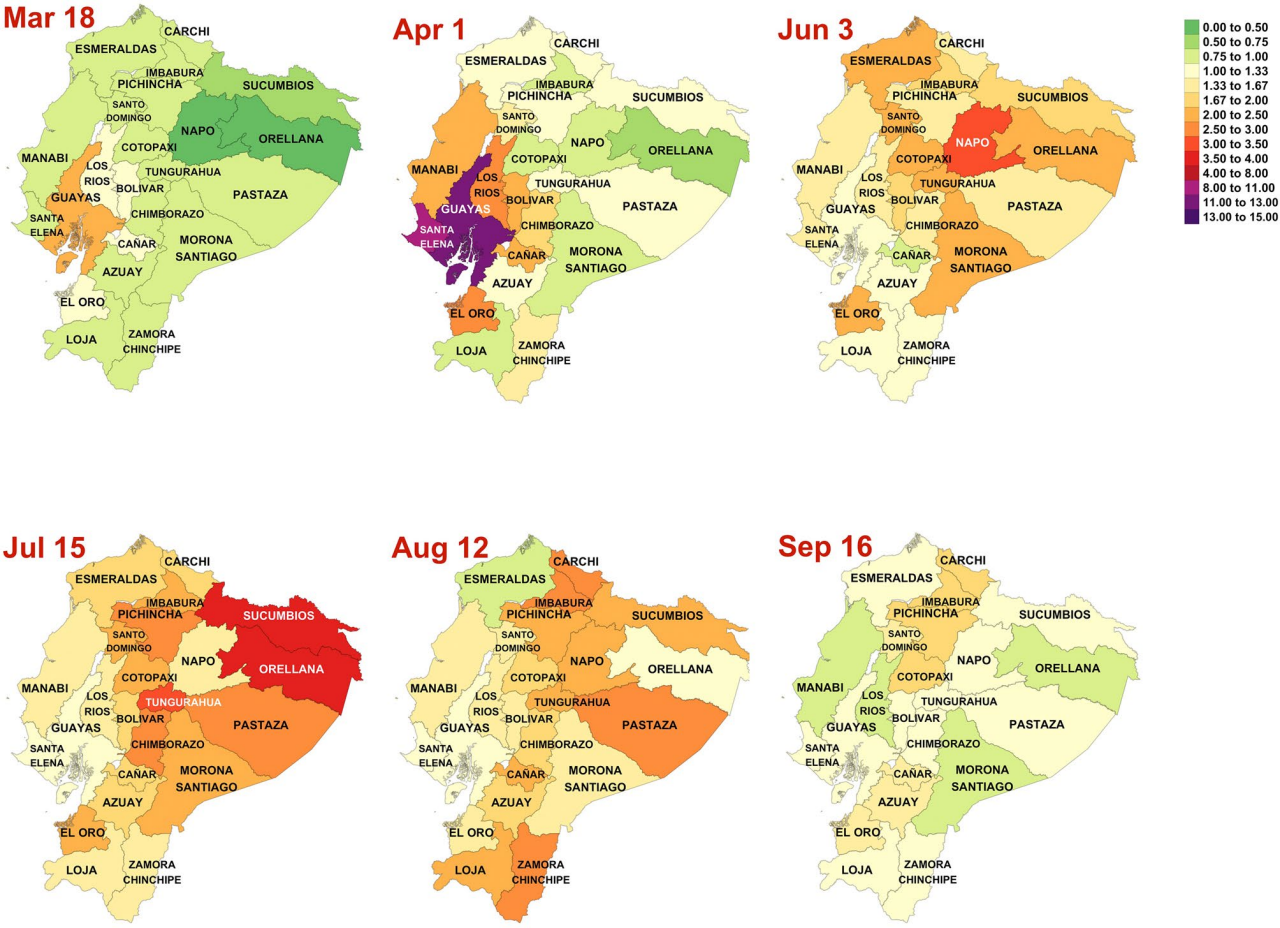
Geographical and temporal evolution of weekly excess death factor for Ecuador. Figure created in R version 4.0.3 (https://www.r-project.org).

Figure 4 visualizes the geographical spread of the EDF aggregated by sex and all age groups, for six selected weeks: the week of March 18, when the lock started, the week of April 1st , in the middle of the lockdown, the weeks of June 3rd, July 15 and August 12, after the lockdown, and the week of September 16, the last week we had data. A figure for all the weeks is provided in the appendix. The figure shows how the hotspot of the epidemic is moving through Ecuador, with different provinces becoming the weekly hotspot at different times.

**Figure 5:**
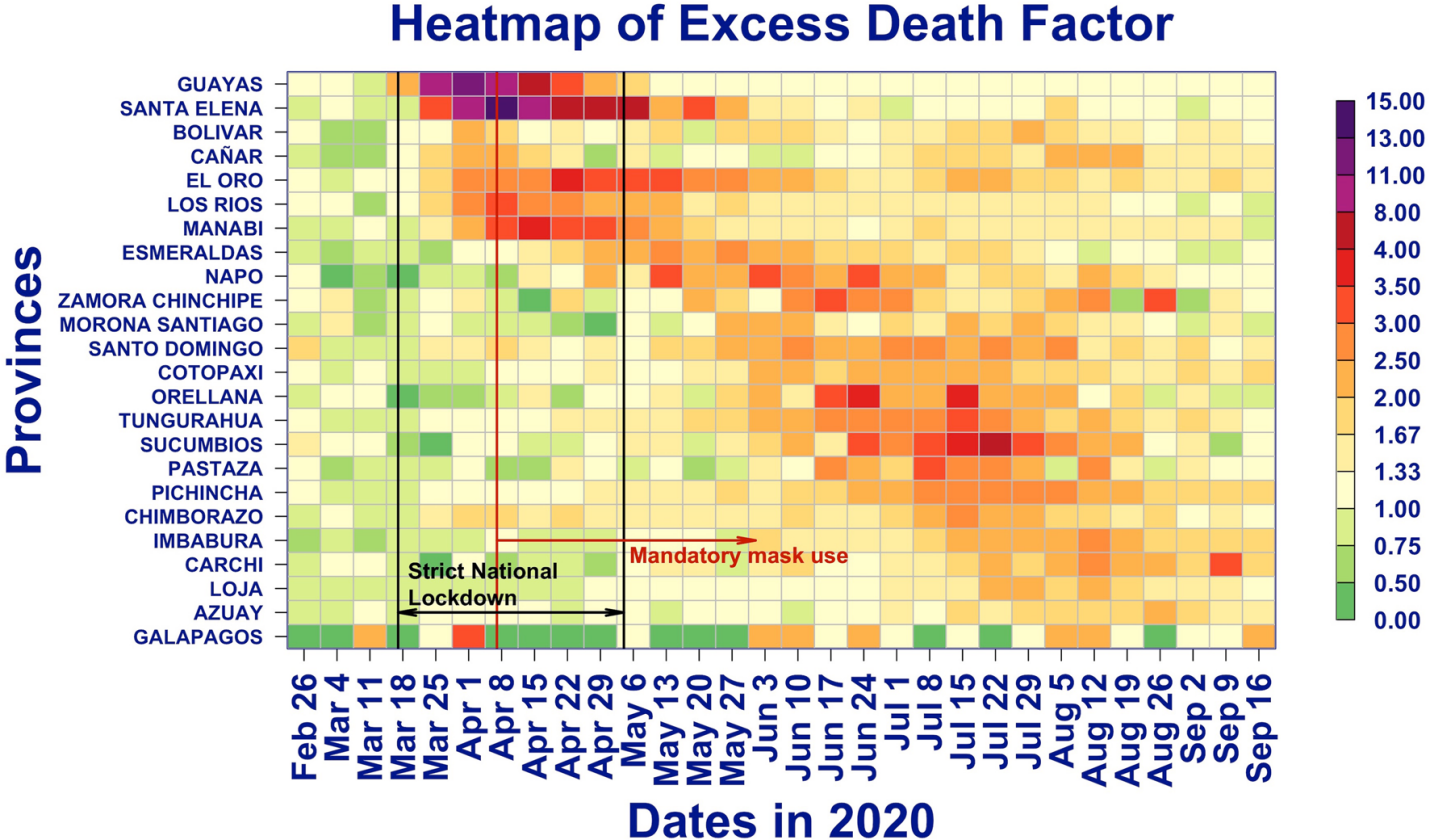
Heatmap of excess death factor by province with national restrictions.

It is easier to visualize the dynamic of the EDFs by displaying as a heatmap (Fig. 5) the time series of EDF. The provinces are ordered by the date of the first time a province recorded a doubling in the number of deaths, i.e., has an EDF of two. The box indicates the period of strict lockdown. The plot clearly shows that COVID-19 did not hit uniformly Ecuador, and that the epidemic exhibits both a temporal and geographic pattern. Interestingly, each province shows a peak period of excess death of about a month or two, during which there are over 2 times more deaths than expected. After that peak period, the excess death ratio declines, but typically remains larger than one. The two vertical black lines indicate the start and end of the strict lockdown, and the red line the start date of mandatory mask use.

There is a delay of several weeks between the time of infection and the time of death. As a result, for some provinces such as Guayas and Santa Elena, the lockdown occurred too late in the course of their outbreak to impact the time and magnitude of the peak of excess deaths. Other provinces, such as Santo Domingo de Los Tsachilas and Pichincha benefitted from the strict lockdown as they achieve their peak well after the strict lockdown was lifted. Finally, some provinces, such as Manabí, achieved their peak in the second half of the strict lockdown and possibly benefitted from it.

### Geographical distribution of mobility

**Figure 6:**
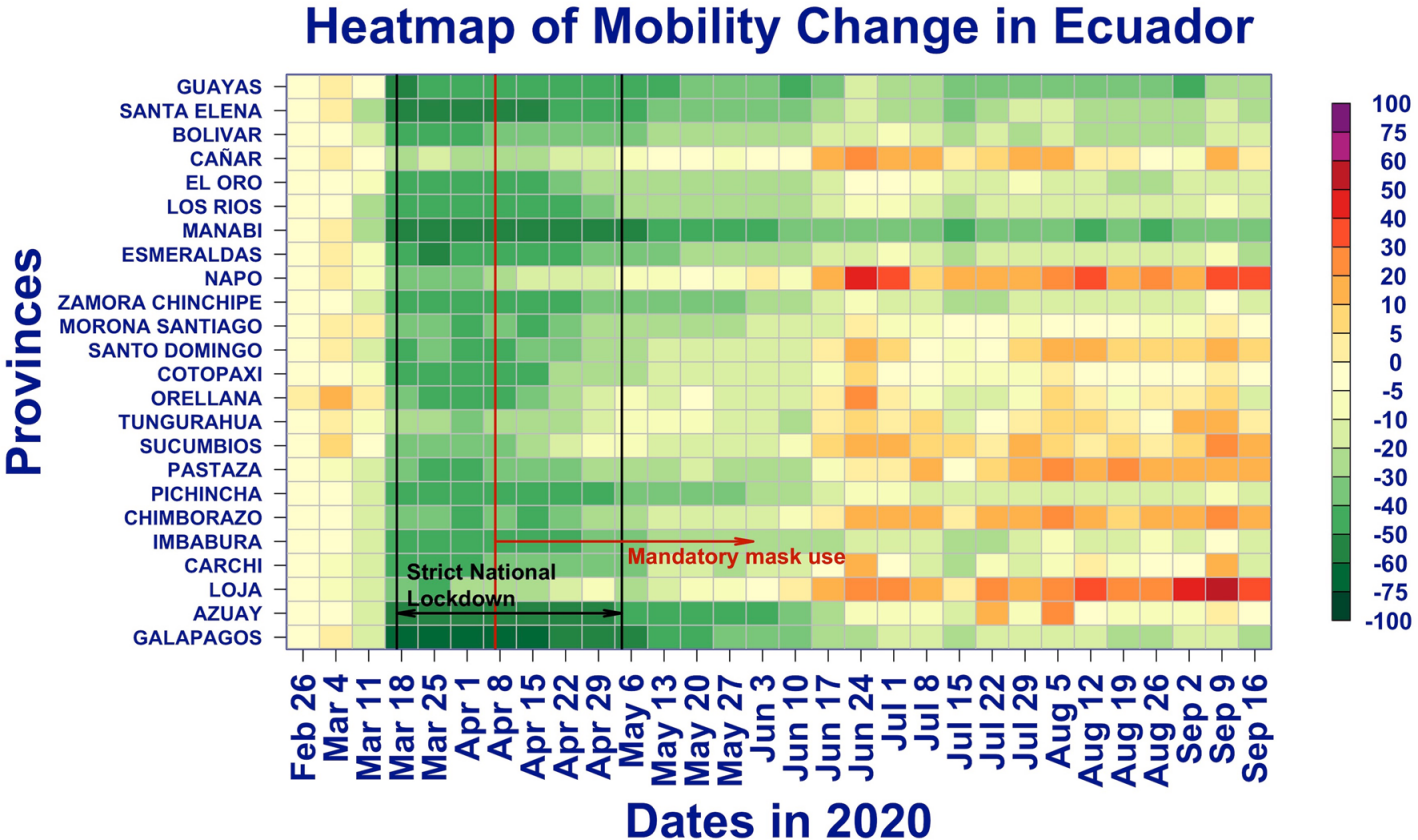
Heatmap of mobility change by province with national restrictions.

Figure 6 shows a similar heatmap for the median mobility score. The two black lines indicate the date of the beginning and end of the strict lockdown. The red line is the date of the mandatory mask order. The plot shows that all the provinces decreased their mobility during the lockdown period and exhibit some increased mobility thereafter. After the lockdown, the population in some of the provinces, such as Guayas, Pichincha, and Manabí, maintained a lower mobility than before the lockdown, while the population in other provinces, such as Bolivar and Napo, the mobility returned to, or even exceeded, the level prior to the lockdown.

### Relating excess deaths to mobility

**Figure 7:**
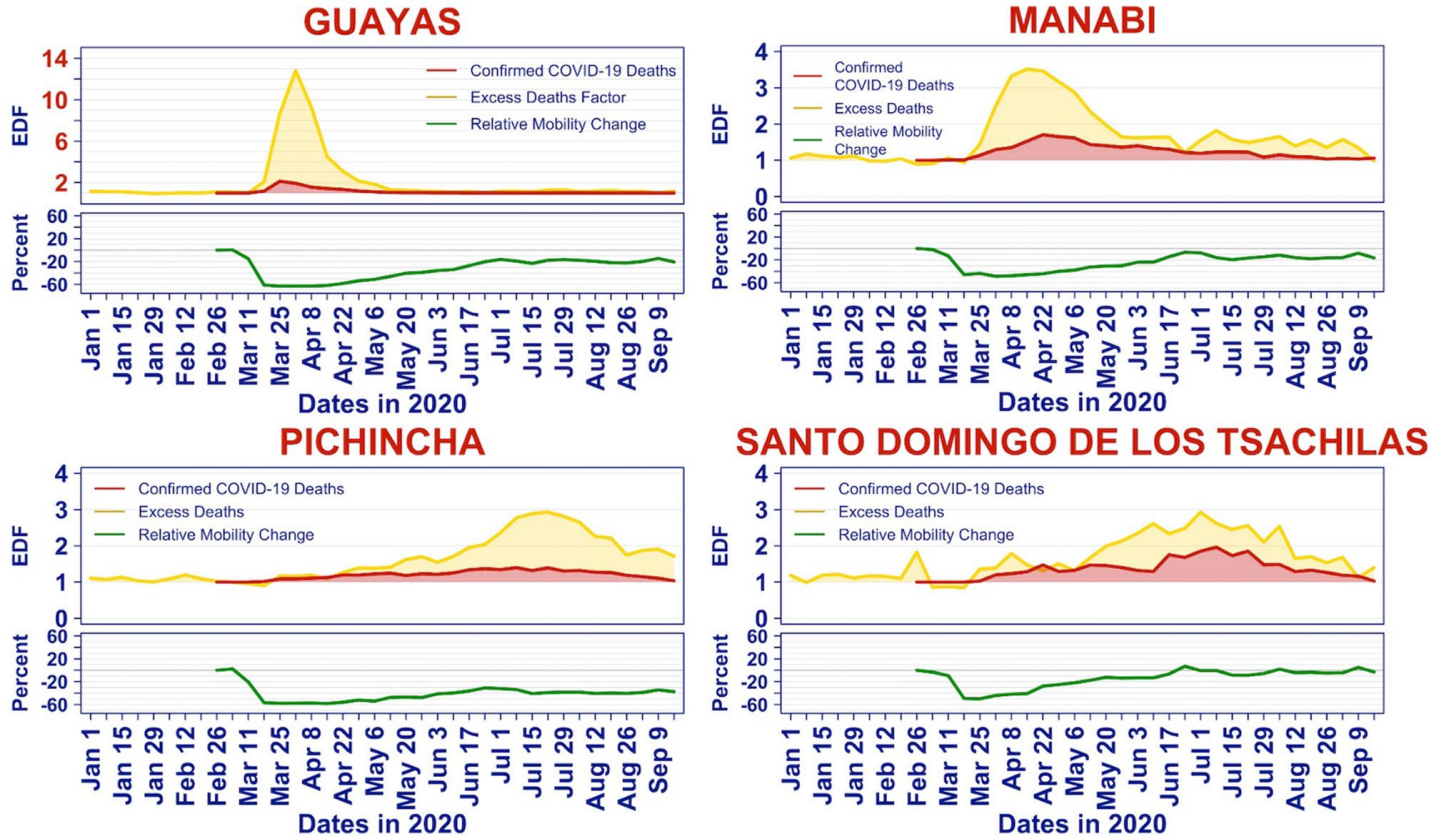
Time series of excess death factor, confirmed COVID-10 deaths, and median mobility.

Figure 7 displays simultaneously the EDF and the median mobility for Guayas, Manabí, Pichincha and Santo Domingo. These four provinces were chosen because they are archetypes of when they reached their peak of EDF with respect to the lockdown period. For Guayas, the lockdown started too late to impact the EDF peak, for Manabí, the lockdown had likely a partial effect on the timing and magnitude of the peak, and both Pichincha and Santo Domingo are examples of peaks occurring well after the lockdown ended. The difference between these two provinces is the pattern of population mobility. In Pichincha, the population maintained a lower mobility than prior to the lockdown, whereas in Santo Domingo, the population mobility returned to pre-lockdown levels. Figure 7, which shows simultaneously the time series of EDFs (in yellow), fraction of deaths attributed to COVID (red) and median mobility score (green), reveals that there is no general pattern linking mobility score to excess deaths that holds across all provinces. As a result, we performed a separate statistical analysis for each of the 24 provinces, and then compared the results in terms of the stage of the outbreak each province was when interventions were implemented.

#### Cross-correlation analysis

**Figure 8:**
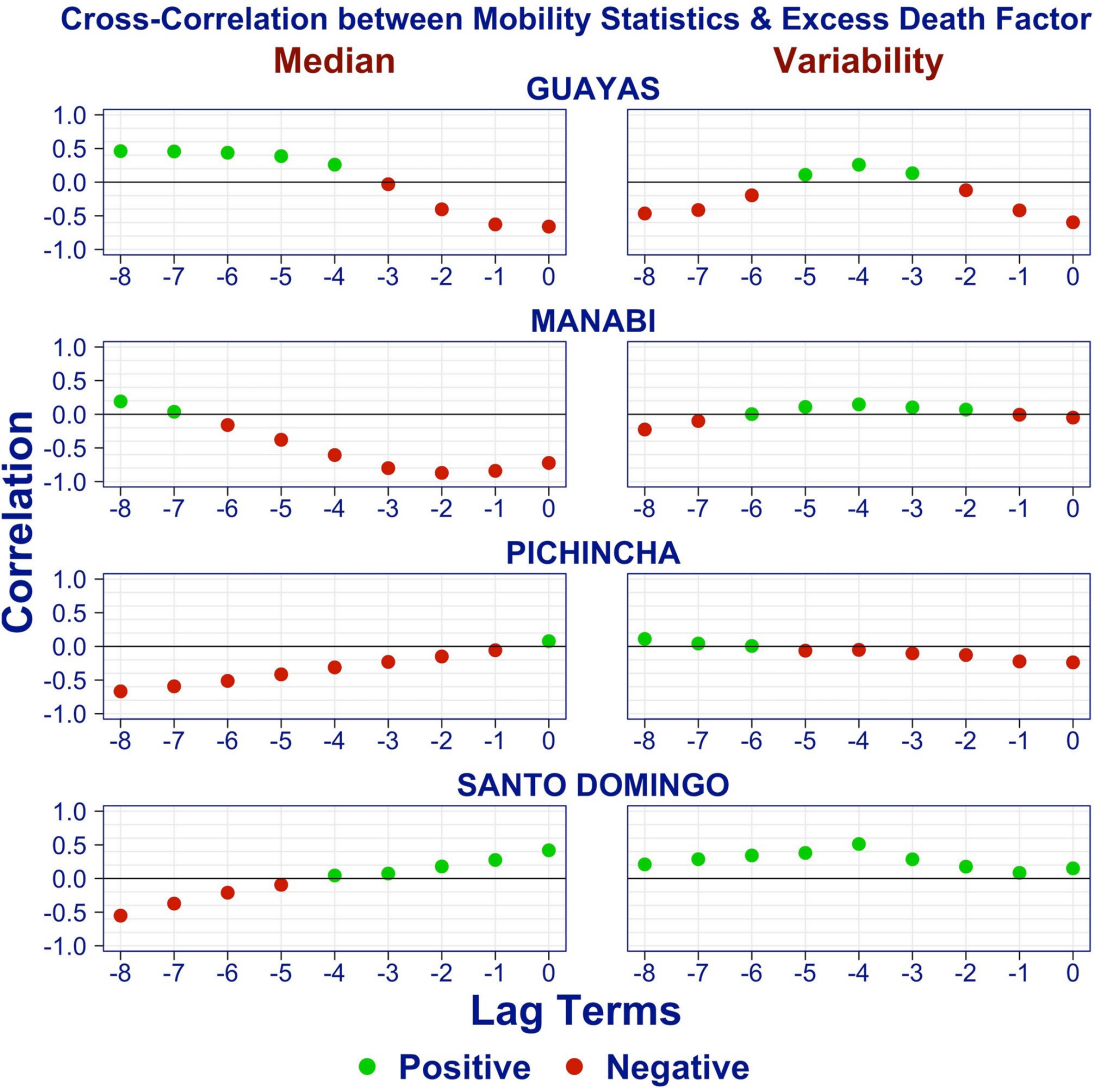
Cross-correlation between time series of excess death factors and mobility statistics.

Figure 8 displays the cross-correlation between the time series of EDFs and mobility statistics for selected provinces: Guayas, Manabí, Pichincha, and Santo Domingo. The x-axis represents the lag, the difference in weeks, between the mobility statistic and the EDF. A lag of zero (right most point) means the correlation is taken between EDF and the mobility statistic in the same week, whereas a lag of -2 corresponds to a correlation between EDF and the mobility statistic for two weeks prior. The colors indicate positive (green) and negative (red) correlations.

In two of these provinces, Guayas and Manabí, the median mobility scores from the past three weeks are negatively correlated with the current EDF. Looking at Fig. 7 we see why: the mobility (green curve) decreases when the EDF (yellow line) is rising. This is indicative of what we expect when a shutdown is instituted because of rising cases.

In contrast, the correlation between EDF and median mobility in the past four weeks is positive in Santo Domingo. Again, Fig. 7 tells the story: observe that in Santo Domingo, after the strict lockdown was lifted, the median mobility slowly returned to its pre-lockdown level. At the same time, EDF increased. Again, this is the pattern one expects if one lifts mobility restrictions prior to having fully contained the outbreak.

Finally, in Pichincha, the cross-correlation is capturing a situation that lies between the previous cases. After the lockdown ended, the mobility slowly increased, allowing the outbreak to take hold. As it became evident that Pichincha was in the grip of an outbreak, mobility reduced slightly.

Looking at the plot of the cross-correlation for the median in the Appendix, we see the described pattern repeat for all provinces, dividing them into provinces whose outbreak was already in progress at the time of the lockdown, and those whose outbreak emerged after the lockdown.

The interpretation for the variability statistics is harder. Looking at cross-correlation plots for the variability for all the provinces (see Appendix), we note that for many provinces, the cross-correlation is positive. This makes intuitive sense: larger variability implies that some (but not all) individuals have mobility. For a very infectious virus like SARS-COV-2^42^, a small fraction of individuals who move about and can transmit the disease suffices.

#### Regression analysis

The regression analysis improves upon the cross-correlation in that it allows to control for age group and sex, considers the joint behavior of a set of explanatory variables, and allows model selection. The latter helps identify which explanatory variables are statistically significant.

We used a log-linear Poisson regression to predict the weekly number of deaths divided into age group and sex categories. By including the logarithm of the predicted death from the baseline model fitted on historical death data as a fixed offset, we can interpret the regression as a model for the excess death ratio. Our model used age group and sex and their interaction as covariates. The inclusion of the age-sex interaction allows the model to fit different death rates for men and women in the same age group. In addition to the demographic variables, we included the value of the median mobility and IQR from the past two to four weeks (names lag 2, lag 3 and lag 4). We applied stepwise regression using the AIC criteria to select which variables are statistically important.

We did not include mobility statistics from the same week or from the one week before to help with the interpretation. Indeed, the mobility statistics of the current and past weeks are correlated, and our model selection could identify those variables as statistically significant, even though we know from the disease progression, and how mobility impacts the spread of disease, that these variables are not causative.

The table of estimated coefficients are provided in Table 2 of the appendix. To help interpret, we prefer to present in Table 1 the multiplier for the expected EDF from a 10% decrease. For example, a 10% decrease in the median mobility two weeks prior in the province of Santo Domingo, reduces the expected EDF by a multiplier of 0.905.

**Table 1:** Multiplier for the expected excess death factor from a 10% decrease of mobility statistics Lags.

Jurisdiction	Median			IQR			% Var. explained
	Lag 2	Lag 3	Lag 4	Lag 2	Lag 3	Lag 4	
GUAYAS	1.713	0.804	0.803	0.803	1.342	0.854	80%
SANTA ELENA	1.448	1.174	0.825	1.078	0.799	0.786	81%
BOLIVAR	1.048					0.875	32%
CANAR	1.117		0.914	0.859			28%
EL ORO	1.182		1.053			0.972	88%
LOS RIOS	1.183					0.885	80%
MANABI	1.240	1.075	0.950	0.946	1.057		82%
ESMERALDAS			1.192			0.914	72%
NAPO	0.831	1.232		0.897	0.846	0.908	57%
ZAMORA CHINCHIPE						0.906	18%
MORONA SANTIAGO	0.885					0.832	38%
SANTO DOMINGO DE LOS TSACHILAS	0.905		1.074			0.751	45%
COTOPAXI	0.914		1.069			0.938	38%
ORELLANA	0.803		1.281		0.904	0.909	35%
TUNGURAHUA		0.829	1.190	0.916		0.765	52%
SUCUMBIOS	0.887						25%
PASTAZA		0.786	1.257	0.845		0.807	39%
PICHINCHA	0.945	0.834	1.289	1.106	0.932	0.856	29%
CHIMBORAZO				1.092		0.868	29%
IMBABURA	0.919			0.899	0.890	0.905	72%
CARCHI		0.907	0.913			0.921	46%
LOJA		0.956				0.855	63%
AZUAY	0.929		1.043		0.915	0.887	25%
GALAPAGOS	1.322	0.754					14%

The empty cells in the table indicate variables that have not been selected. For example, the regression model to predict the EDF in the province of Esmeralda only includes the median mobility score from the four weeks before. The provinces are ordered according to the first date that the EDF was two.

Again we see two groups: the provinces for which multipliers of a 10% mobility reduction in lag 2, or when lag 2 is not significant, in lag 3, is greater than one or is less than one. As we observed in the correlation analysis, the provinces that had early peaks during the strict shutdown all show a factor that is greater than one. Indeed, these provinces had an increase in the weekly EDFs and a decrease in mobility at the same time.

The second group had their peak after the end strict shutdown. As we noted the median mobility was increasing during that time. And as mobility increased so did the EDFs. In contrast, multipliers less than one represent the effect of a 10% decrease in mobility.

We note that for almost all provinces the variability (IQR) four weeks prior that is statistically significant, a 10% decrease in the IQR leads to a reduction in the expected EDF.

Finally, the table presents the measure of variability explained by the mobility statistics defined in the Methods section.

A limitation of our analysis is that it does not account for the dynamic of the outbreak. We plan to present such an analysis in a forthcoming paper.

## Discussion

This papers contributes to our understanding of non-pharmaceutical interventions (NPI) by probing the relationship of macroscopic mobility patterns and excess death. We made three key discoveries: (1) excess deaths spread across the country in a west-to-east gradient with the epicenter focusing on Guayas, (2) lockdowns lead to a large reduction in local, individual-level mobility, and (3) both the median and variability of mobility indices are reasonable predictor variables of excess deaths although the relationship between mobility and excess deaths is complicated. Based on these observations, it is conceivable that local lockdowns should be implemented in a more targeted manner: immediately for provinces that experience on-going COVID-19 outbreaks to prevent large magnitude of excess deaths; and delayed for provinces that are geographically far away from the epi-center of the outbreak. This approach could strike a more overall favorable balance between infection control and the social disruption caused by lockdowns.

The complex relationship between mobility and excess deaths is illustrated by the dynamics in Guayas, where the epidemic was already a significant cause of mortality when the national lockdown orders were issued; in this context, the effect of reduced average mobility might have a reduced effect as enough people are infected to sustain transmission even given reduced contact rates. However, in regions where the lockdown occurred before there was significant excess death, the slow increase in mobility corresponded to an increase in EDF. While exact causation is difficult in these studies, our study is consistent with the idea that NPIs are partially mediated though reduced mobility and that those effects are strongest when the endogenous transmission rates are low within a given region due to a limited number of infected persons.

Mobility can serve as an indicator of the extent of SARS-CoV-2 transmission in a community. However, its utility is limited by several factors. First, the relationship between mobility and excess deaths depends on the context of the epidemic and government response, as demonstrated by our analysis in this work. For example, we found that the mobility measure negatively correlates with EDF with a two-weeks lag for provinces (e.g. Guayas) experiencing the first excess deaths wave in April and May 2020. This negative correlation is because of implementation of lockdown while increasing deaths as a result of wide-spread SARS-CoV-2. On the other hand, we found positive correlations between the two in provinces (e.g. Santo Domingo) that did not experience large outbreaks during the first wave of excess deaths. Presumably, this is because as lockdown is relaxed, increases in mobility led to increases in excess deaths. Second, we and others^11,12^ often use a single or a couple of measures (median or variability) of mobility; however, the spread of SARS-CoV-2 may be impacted differently by different types of mobility. For example, it was suggested long-distance travel may help the dispersal of the pathogen^11,43^ In addition, mobility measures often summarize travels of different types, i.e. grocery shopping, travel to work, go to park etc. These different types may contribute to SARS-CoV-2 spread differently.

## Conclusion

This paper presents the Excess Death Factor (EDF) time series for all provinces in Ecuador, and relates them to mobility data derived from cellular phone data that was obtained from GRANDATA and the United Nations Development Program for the period starting on March 1st, 2020 and ending on September 23rd, 2020. The data reveal that the provinces were hit by the pandemic in a clear spatio-temporal pattern, with the peak EDF moving across Ecuador over time in a relatively short six-month period. A statistical analysis reveals that the relationship between human mobility and EDF show two archetypes, one pattern for the provinces whose peak EDF occurred during the strict lockdown, and another pattern for the provinces who reached the peak of their EDF after the conclusion of the strict lockdown. Finally, we demonstrate both the median mobility and the variability of mobility as measured by the IQR are statistically significant predictors for the EDF.

## Supplementary Information

Supplementary Information.

## Data Availability

Researchers interested in the mobility data should contact GRANDATA. The historic mortality data (2015–2019) is publicly available [https://www.ecuadorencifras.gob.ec/defunciones-generales] but the 2020 COVID-19 mortality data cannot be released without permission from the Ecuador Ministry of Health.
